# Perspectives from Nurse Managers on Informatics Competencies

**DOI:** 10.1155/2014/391714

**Published:** 2014-03-23

**Authors:** Li Yang, Dan Cui, Xuemei Zhu, Qiuli Zhao, Ningning Xiao, Xiaoying Shen

**Affiliations:** ^1^School of Nursing, The 2nd Affiliated Hospital of Harbin Medical University, Harbin Medical University, 246 Xuefu Road, Harbin, Heilongjiang 150086, China; ^2^Department of Emergency, The 2nd Affiliated Hospital of Harbin Medical University, Harbin Medical University, Harbin, Heilongjiang 150086, China

## Abstract

*Background and Purpose*. Nurse managers are in an excellent position for providing leadership and support within the institutions they serve and are often responsible for accessing information that is vital to the improvement of health facility processes and patients' outcomes. Therefore, competency in informatics is essential. The purposes of this study are to examine current informatics competency levels of nurse managers and to identify the variables that influence these competencies. *Methods*. A questionnaire designed to assess demographic information and nursing informatics competency was completed by 68 nurse managers. Multiple linear regression analysis was conducted to analyze the factors influencing informatics competency. *Results*. Descriptive analysis of the data revealed that informatics competency of these nurse managers was in the moderate range (77.65 ± 8.14). Multiple linear regression analysis indicated that level of education, nursing administration experience, and informatics education/training were significant factors affecting competency levels. *Conclusion*. The factors identified in this study can serve as a reference for nurse managers who were wishing to improve their informatics competency, hospital administrators seeking to provide appropriate training, and nursing educators who were making decisions about nursing informatics curricula. These findings suggest that efforts to enhance the informatics competency of nurse managers have marked potential benefits.

## 1. Introduction

Since the introduction of computers in healthcare settings, the expected competency of nurses who work with information and communication technologies has been under review. In the past few years, the need for informatics competency has grown, driven by the use of technology in clinical practice. Informatics competency was defined as “the integration of knowledge, skills, and attitudes in the performance of various nursing informatics activities [1].” The importance of informatics competency for all nurses was articulated in the 2001 American Nurses Association's (ANA's) Scope and Standards of Practice for Nursing Informatics. This document divided informatics competency into three general categories: computer literacy skills, information literacy skills, and overall informatics competency [2].

In 2006, the Technology Informatics Guiding Education Reform (TIGER) Summit introduced a vision of nurses using informatics in practice and education to support patient safety and offer high quality care. The resulting TIGER action plan for healthcare delivery organizations included a recommendation to develop a professional nursing model that supports the attainment and development of competency in informatics. The TIGER recommendation is to (1) allow informatics tools, principles, theories, and practices to be used by nurses to make healthcare safer, effective, efficient, patient centered, timely, and equitable and (2) interweave enabling technologies transparently into nursing practice and education, making information technology the stethoscope for the 21st century [3, 4].

The necessary informatics competencies for nurses and nursing students have been studied in several projects. For example, in 2002, Staggers et al. published the results of a Delphi study that identified core informatics competencies for nurses at four levels of practice: [5] beginning nurse, experienced nurse, informatics specialist, and informatics innovator. Staggers' study was the first research-based master list of informatics competencies for nurses to span four levels of nursing practice and cover competencies in computer skills, informatics knowledge, and informatics skills; many researchers have used this master list. Curran [6] added competencies related to evidence-based practice and extracted items from the master list to propose a list of nurse practitioner competencies. Ornes and Gassert [7] used the master list to determine if informatics competencies were included in the curriculum of a baccalaureate program.

Another research on informatics includes a survey of graduating baccalaureate nurses, the results found that information technology skills were in moderate level [8]; this study also revealed that the students were most confident in their Internet, word processing, and systems operations skills and least confident in care documentation and planning, valuing informatics knowledge, skills development, and data entry competencies. Pravikoff et al. [9] provided useful insights into the state of information literacy in 760 US clinical nurses. The authors found that 82% did not use the hospital library, 72% had not evaluated research reports in the last year, approximately 60% never searched CINAHL and MEDLINE, and 77% had never been trained to search bibliographic databases. The study concluded that RNs in USA were not ready for evidence-based practice because of the gaps in their information literacy and computer skills, limited access, and attitudes toward research.

Most studies of informatics competency involve nurses or students; there are few studies addressing the informatics competencies of nurse managers in clinical practice. One of the few studies that focused on nurse managers was conducted by Hart [10] in 2010. Based on the research of Staggers et al., Hart performed a three-round Delphi study to determine core informatics competencies for generic nurse managers that resulted in a list of 49 core informatics competencies.

At present, the use of nursing informatics has just begun in China; nursing informatics education lags behind other countries and the core informatics competencies for nurses or nurse managers in mainland China have not been established. Therefore, the purpose of this study was to investigate the level of informatics competencies of nurse managers and examine the influencing factors.

## 2. Methods

### 2.1. Participants

The convenience sample included volunteer nurse managers in a general hospital affiliated with Harbin Medical University. In October 2012, we sent questionnaires to every nurse manager (*N* = 75). Participants were told the aim of the study and that participation was voluntary, their anonymity would be protected, data would be handled with confidentiality, and they could withdraw from the study at any time. Names were replaced by code numbers upon return of the completed questionnaires to assure anonymity, and the completed questionnaires were examined by only the study investigators.

Sixty-eight valid questionnaires were returned (90.67%) and sixty-seven of the respondents were female. The demographic data of the participants was shown in Table 1. Over one-half of the participants (58.8%) were below 40 years old, and 35.3% held master's degrees. Some respondents had worked at the Second Affiliated Hospital of Harbin Medical University for more than 20 years (45.6%), and the same percentage had been in an executive position for less than five years. 55.9% respondents had not received education or training of informatics knowledge and skills.

### 2.2. Measure

The first part of the questionnaire consisted of queries about the general characteristics of the respondents, including the respondents' gender, age, highest level of education, job experience, and years of experience in nursing administration. The second part investigated nurse managers' informatics competencies using 49 informatics competency items identified by Hart in three categories: computer skills (25 items), for example, using e-mail and using the Internet to locate and download items of interest; informatics knowledge (20 items), such as recognizing the use and/or importance of nursing data for improving practice and identifying the basic components of the current computer system; and informatics skills (4 items), for example, performing basic trouble-shooting in applications.

The 49 competencies were translated and reviewed for relevance by an expert panel of four administrative nurses skilled in informatics. Discussions between the researchers and the author of the original questionnaire were held to verify the cross-cultural equivalence of the Chinese version. Respondents rated their personal skill level on each competency item using a five-point Likert-type scale: 1 = no experience or no competence, 2 = minimal skills, 3 = some or moderate skills, 4 = good skills or competence, and 5 = expert skill or fully competency. The final standard score was final score/highest possible score ∗100%.

### 2.3. Ethical Considerations

The protocol for this study complies with the Helsinki declaration and was approved by the appropriate committees and officials. The study was briefly explained to potential participants and they were informed that involvement was completely voluntary and that they could withdraw at any time with no negative impact.

### 2.4. Statistical Analysis

Statistical analysis was performed using the software package (SPSS 13.0, Beijing, China). Continuous variables are expressed as mean ± SD; categorical variables are presented as frequencies. We performed three sets of analyses. First, we calculated Cronbach's *α* to establish the internal consistency reliability of the questionnaire in our study population. Second, we conducted ANOVA to assess the differences among computer skills, informatics knowledge, and informatics skills. Third, a multiple linear regression analysis was used to assess the independent effects of demographic variables on informatics competencies. A two-tailed *α*-value of 0.05 was set for significance.

## 3. Results

### 3.1. Instrument Validity and Reliability

The content validity of the questionnaire was approved by five experts who were experienced in both nursing and information management. To confirm the validity of the questionnaire, we invited ten nurse managers to participate in a pretest to ensure that the items were easily understood. Cronbach's *α* values were higher than 0.85, which indicated high internal consistency.

### 3.2. Informatics Competencies

**Figure 1:**
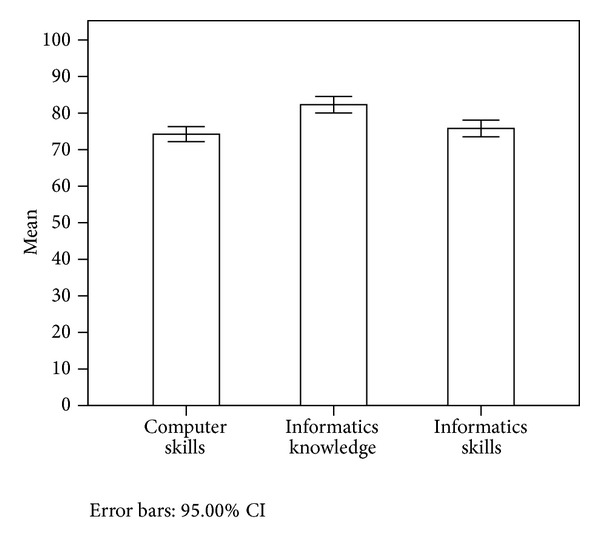
Difference between three domains of informatics competencies. Informatics knowledge was significantly higher than computer skills and informatics skills (*P* < 0.000). However, computer skills were not different from informatics skills (*P* = 0.313).

The standard score of informatics competencies for all respondents was 77.65 ± 8.14, which represents a medium level of informatics competency. The scores on informatics knowledge were significantly higher than computer and informatics skills scores (*P* < 0.05). However, the difference between computer and informatics skills scores was not significant (*P* > 0.05) (Table 2 and Figure 1).

### 3.3. Multiple Linear Regression Analysis Models

To understand the influencing factors of informatics competencies, multiple linear regression analysis was conducted. The overall model showed a statistically significant relationship. Education level, experience of nursing administration, and informatics education/training accounted for 71.2% of total variability in informatics competencies (Table 3).

## 4. Discussion

Nurse managers, as administrators within their hospital or health facility, need the knowledge and skills to use databases and information systems for the collection and interpretation of statistical data on patient care, including length of stay, staff rosters, patient acuity/dependency, budgets, and resource ordering [11]. Information competency is essential for managers, and it will become more important in the future as more sophisticated clinical information techniques are developed.

In the present study, we found that education level had a significant impact on informatics competencies. One possible reason for this relationship is that those with higher education levels have learned information retrieval and evidence-based nursing systematically. For example, the preparation of a master's thesis requires proficiency in technological skills, including keyboarding, word processing, multimedia presentations, online literature searches, e-mail correspondence, and use of the Internet, which cover many of the informatics skill items in this study.

The results also revealed that a nurse manager's computer and informatics skills were lower than his/her informatics knowledge. Computer skills encompass the use of computer hardware and software and allow for basic technological interface (i.e., the use of e-mail and the Internet, online literature searches, and use of application, such as word processing). Informatics skills are the use of methods, tools, and techniques particular to informatics, while informatics knowledge is the theoretical and conceptual basis for the specialty. For example, informatics skills include techniques and tools used in systems analysis and project management, while informatics knowledge includes familiarity with nursing classification and reasons for systems slowness [5]. The low scores in computer skills and informatics skills should be increased through education or training.

The results indicate that respondents' information related to education or training had a significant impact on their overall informatics competencies. However, more than one-half of the respondents (38) lacked any informatics knowledge and skills education in school or at work. This finding suggests that providing more informatics courses and training in school and at work is important. Academic courses in computer skills, informatics skills, evidenced-based practice, effectiveness research, and nursing administration or operational business management, including a required course in nursing informatics for nurses preparing them for an administrative career, could be developed in tandem with continuing education opportunities offered by academic or health institutions.

Nursing administration experience is a factor that negatively affected informatics competencies scores in this study. In other words, more administrative experience was associated with a lower level of informatics competencies. This is closely connected with China's national conditions, and there are several possible explanations. The respondents with more administrative experience are from an era when computers were used less frequently in school or at work, trained at a secondary level vocational school (the primary training site for nurses in China before 2000), or were affected by conventional thinking and traditional views as their administrative experience grew. Therefore, nurse managers with administrative experience should be targeted for computer and informatics continuing education, which will allow them keeping pace with the rapid development of information technology, particularly in the field of patient safety and care.

### 4.1. Limitations

There are several limitations to this study. The participants were not randomly selected; therefore, the respondents may not adequately represent the population of interest. The other limitation is that the informatics competencies data was collected through a self-report questionnaire. Consequently, nurse managers could under- or overestimate their informatics competency. However, since the self-ratings were not considered in a performance appraisal, there was little motivation to overestimate. To objectively assess informatics competencies, future studies should use other tools to measure the true level of the participants' informatics competency.

### 4.2. Conclusion

In a field where technology changes on a daily basis, it is crucial for nurses and nurse managers to stay up-to-date on trends and information [12]. The results of this study confirmed that education level, experience in nursing administration, and information education/training were significant factors affecting informatics competency. Nursing informatics knowledge and skills are essential in modern nursing education (initial and continuing education programs) to ensure that nurses are proficient in technological and cutting-edge clinical applications [13]. Thereby, the nurses and nurse managers are able to offer the highest levels of patient care and safety.

## Figures and Tables

**Table 1:** Characteristics of research participants (*N* = 68).

Variable	*N*/Mean	%/SD
Gender		
Female	67	98.5
Male	1	1.5
Mean age (years)	39.88	5.57
Age categories		
≤40	40	58.8
>40	28	41.2
Education		
Bachelor degree	44	64.7
Master degree	24	35.3
Job experience (years)	19.68	6.56
Job experience categories		
≤10	9	12.8
11~20	27	38.6
≥21	32	45.6
Experience of nursing administration (years)	7.26	4.66
Experience of nursing administration categories		
≤5	32	45.6
6~10	21	30.0
≥11	15	21.4
Related education or training		
Yes	30	44.1
No	38	55.9

**Table 2:** Informatics competencies of nurse managers.

Item	Mean	SD
Total informatics competencies	77.65	8.14
Computer skills	74.24	8.44
Informatics knowledge	82.29	9.35
Informatics skills	75.81	9.37

**Table 3:** Multiple linear regression analysis of predicted variable on informatics competencies (stepwise).

Model	Variable in the equation	*B*	SE	Beta (*β*)	*P*	*R*	*R* ^2^	*P*
Total Informatics Competencies	Constant	53.746	3.946		0.000	0.844	0.712	0.000
	Education level	9.048	1.138	0.535	0.000			
	Experience of nursing administration	−1.097	0.118	−0.628	0.000			
	Information education/training	3.870	1.113	0.234	0.001			

Note: Multiple linear regression analysis is regression coefficient, SE is standard error of coefficient, *β* is standardized regression coefficient, *R* is multiple correlation coefficient, *R* ^2^ is proportion of variation in dependent variable explained by regression model, *P* is level of statistical significance.
